# Cost-Benefit Analysis and Emission Reduction of Energy Efficient Lighting at the Universiti Tenaga Nasional

**DOI:** 10.1155/2014/745894

**Published:** 2014-07-15

**Authors:** G. S. B. Ganandran, T. M. I. Mahlia, Hwai Chyuan Ong, B. Rismanchi, W. T. Chong

**Affiliations:** ^1^Department of Mechanical Engineering, Universiti Tenaga National, 43000 Kajang, Selangor, Malaysia; ^2^Department of Mechanical Engineering, Syiah Kuala University, Banda Aceh 23111, Indonesia; ^3^Department of Mechanical Engineering, Faculty of Engineering, University of Malaya, 50603 Kuala Lumpur, Malaysia

## Abstract

This paper reports the result of an investigation on the potential energy saving of the lighting systems at selected buildings of the Universiti Tenaga Nasional. The scope of this project includes evaluation of the lighting system in the Library, Admin Building, College of Engineering, College of Information Technology, Apartments, and COE Food court of the university. The main objectives of this project are to design the proper retrofit scenario and to calculate the potential electricity saving, the payback period, and the potential environmental benefits. In this survey the policy for retrofitting the old lighting system with the new energy saving LEDs starts with 10% for the first year and continues constantly for 10 years until all the lighting systems have been replaced. The result of the life cycle analysis reveals that after four years, the selected buildings will bring profit for the investment.

## 1. Introduction

In order to ensure a comfort, safe, and productive environment, the lighting system must provide suitable condition with desired illumination level. At the same time, lighting system needs to be designed in such a way that consumes the optimum amount of energy. In this modernized era, light source can produce the equal light compared with the traditional lighting systems used 20 years ago, while consuming half the energy input. Malaysia as a fast developing country has to look forward to energy efficiency technologies due to several factors such as cost increment in building new power plants, continuing shortfall between electricity demand and supply, and competing needs for investment capital.

Among all the electric consumers, lighting has one of the highest shares in the residential and commercial sector. Lighting accounts for approximately 20% to 30% of the electricity consumption worldwide [1, 2]. By switching towards more energy efficient lighting technologies, a considerable amount of energy could be saved [3]. The study by Trifunovic et al. [4] showed a potential energy saving of up to 27% in residential and 30% in the commercial sector.

The process of replacing inefficient light systems with more advanced and high efficiency systems are called lighting retrofits. The success of a retrofit program depends on different parameters, such as policies and regulations, occupant's expectation, building specification, and human factors, which has the highest effect among other parameters [5]. These parameters are highly interdependent and could have a significant impact on the design.

The increase in energy consumption contributes significantly to the environment in consequences of the emission production. Almost 80% of the world electricity is produced from the combustion of fossil fuels. This eventually has changed the pattern of emission production. The gases that are being produced by burning the fossil fuels are “greenhouse gases” which contributes to global warming, ozone depletion, acid rain, and other negative impacts [3].

### 1.1. Incandescent Light Bulbs

Incandescent light bulbs have been the most commonly used light sources over the past one and a half decade also called the “Edison Bulb.” They have a simple technology; when connected to a power supply, the electric current heated the wires and tungsten filament to 4,000 degrees Fahrenheit and tungsten begins to evaporate. Without the inert gasses (argon and nitrogen), the tungsten particles would collect on the inside of the glass, causing it to darken. The gasses, however, collect the tungsten particles and send them back to the filament. However, almost 90% of the energy generated by an incandescent bulb is released as heat, not light.

### 1.2. Compact Fluorescent CFL

In the past few years, incandescent bulb starts to give way to more efficient fluorescent (FL), compact fluorescent (CFL), and light emitting diode (LED) lighting systems. CFL bulbs are one of the most successful innovations in the lighting industry; the modern CFL bulbs last 10 times longer than traditional incandescent bulbs. They consume far less energy to produce the same amount of light. For instance, a 15-watt CFL lamp emits the same amount of light as 60-watt incandescent bulb. Despite the great advantages of CFLs they have some limitations. Some CFLs do not perform well in low temperatures; it is possible for a CFL to produce radio frequency interference (RFI); they are not resistant to internal shock, and they have mercury within their glass cover. Many commercial and industrial facilities have old-fashioned inefficient FL lighting systems such as T-12 FL [6].

### 1.3. LED

**Figure 1:**
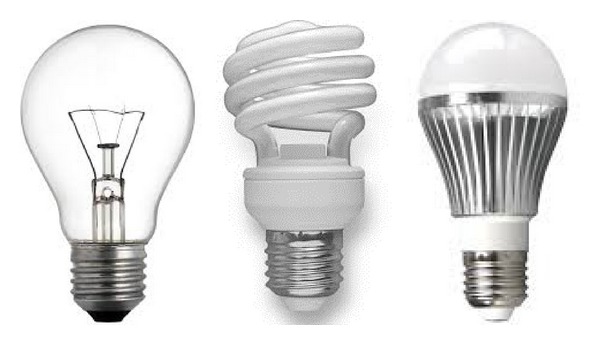
Incandescent, CFL, and LED light bulbs.

The LED is what is called a “solid-state lighting” technology, or SSL. Basically, instead of emitting light from a vacuum or a gas, a SSL emits light from a piece of semiconductor made of a positively charged and a negatively charged component. The light is emitted when electrons move around within the semiconductor structure from the negative to the positive layer. In the early LED models, the structure of the LED causes some of that light to get trapped inside. Therefore, the old models are generally dimmer than an incandescent bulb. However, this problem has been solved in the new models and LED bulbs have brightened up. Many researches are in progress to optimize the performance and light quality of the LED bulbs and at the same time reduce their price [7–9]. There are basically 2 types of LEDs: the 5 mm LED chips and the high-output chip on board (COB). The 5 mm LED has low light output and lacks proper thermal path that is essential for maintaining the LED's junction temperature. Normally the luminous of the 5 mm LED would reduce to half of its original value after 6000 hours. The COB is known as the current choice for lighting since it offers far superior luminous output as well as having proper thermal path for regulating the LED's junction temperature.  Figure 1 shows the Incandescent, CFL, and LED light bulbs.

Several studies have been conducted on the benefits of retrofitting conventional lighting systems with the new low energy ones [10, 11]. Light emitting diode (LED) lamps are more effective than incandescent and CFL bulbs and have a longer lifetime while providing similar luminous. Uddin et al. [12] found that LED lamps are more expedient than conventional bulbs and also advance in terms of environment friendly but economical wise LEDs have higher initial costs. Chen and Chung [13] have studied retrofitting LEDs with T8 fluorescent tubes; they found that by replacing 36 W T8 fluorescent lamp with 20 W LED, would have around $288 saving in 5 year operation.

Ryckaert et al. [14] conduct a research on the ups and downs of retrofitting LED tubes with T8 FL lamps. They analyse twelve different LED tubes and the results show that a one-to-one lamp replacement could lead to an inadequate quantity and quality of illumination of the work plane. In order to address this issue, additional LEDs are required which consequently would decrease the potential energy savings. In another study, Stefano [11] identified three main barriers to the cost effective installation of energy efficient lighting technologies in offices such as low lighting system operating hours, low cost of electricity, and high initial expense of energy efficient lighting components. Vahl et al. [15] analyse the long term sustainability of retrofitting inefficient light bulbs with CFLs and LEDs. They found that generally CFLs have the highest annual cost and toxic waste; FL tubes are the most economical alternative, but if their lifespans shorten and LED prices drop or achieve higher efficiency, LED becomes the most sustainable and economically alternative.

Mahlia [16] shows the method to calculate the potential emission from the fuel mix used by Malaysia to generate electricity. The author uses the polynomial equation to predict the energy consumption and the emission produced in the years 2002 to 2020. The data of per unit of CO_2_, SO_2_, NO_*x*_, and CO emitted for electricity generation was obtained. Another study shows the prediction in a reduction on the amount of CO_2_ and SO_2_ emission in the years 2010, 2020, and 2050 by using biogas energy compared to traditional method [17].

In Malaysia, around 40% of greenhouse gases are contributed by residential buildings [18]. Therefore, it is necessary to reconstruct or retrofit the current buildings according to the green building valuation. In this project, the current lighting systems used at the Universiti Tenaga Nasional have been considered in which most of the current lamps are CFL two pin bulbs and fluorescence tubes. The current lighting system consumes considerable amount of energy to provide required light. The main objectives of this project are to design the proper retrofit and to calculate the potential electricity saving and the payback period for returning the investment. Also the potential environmental benefits have been analyzed as one of the key objectives of the project.

## 2. Methodology

**Figure 2:**
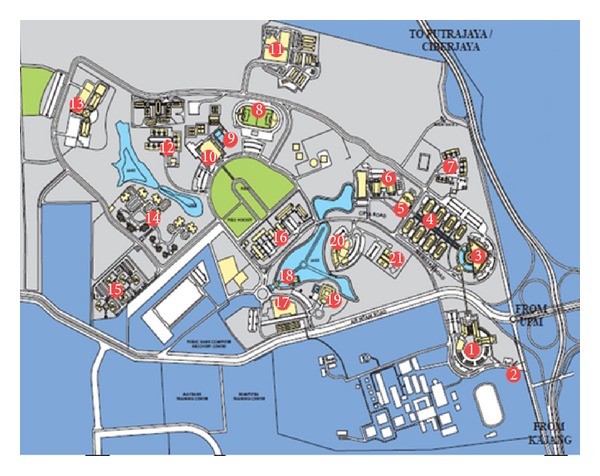
Map of Universiti Tenaga Nasional. (1) Library, (2) Main Gate, (3) Administration Building, (4) Laboratory and Training Block, (5) COE Food court, (6) College of Engineering, (7) Murni Apartment, (8) Mini Stadium, (9) Multipurpose Hall, (10) TNB Equestrian Centre, (11) Cendekiawan Apartment, (12) College of Information Technology, (13) Staff Quarters, (14) Amanah Apartment, (15) Ilmu Apartment, (16) Food court 1, (17) Lake Café, (18) Mosque, (19) Residence Hotel, and (20) Twin Tutorial Hall.

The scope of this project includes evaluation of the lighting system in the Library, Admin Building, College of Engineering, College of Information Technology, Apartments, and COE Food court of the university. The calculation of the energy consumption is based on the wattage input consumed by light bulbs.  Figure 2 shows the location of each building in the compound.

**Figure 3:**
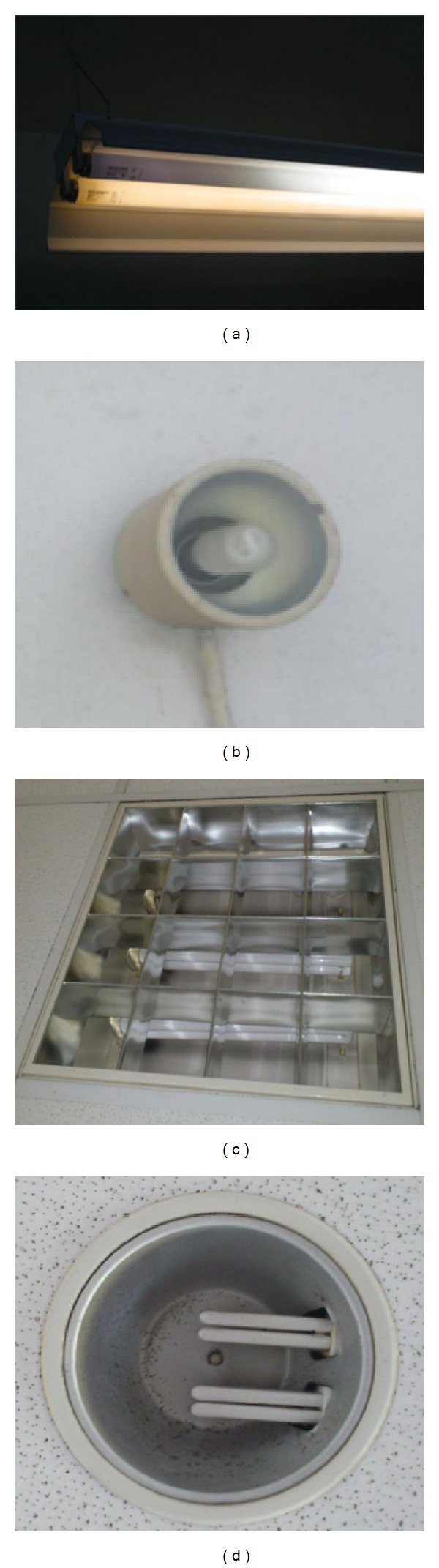
(a) Philips fluorescent tube 36 W 4 feet, (b) Philips compact fluorescent light (CFL) bulb 14 W fitting E27, (c) Philips 36 W/4 Pin fitting 2G11, and (d) Philips PL-C 13 W 2 Pin fitting G24.

The number and type of lighting bulbs were counted manually during a walk-through audit; also the results were double checked with the lighting layout to minimize the data gathering error. The lighting layout was obtained from Facility Development & Management Department and it shows the location and type of light used in each building in details. It has been identified that the most commonly used light bulbs are; Philips compact fluorescent light (CFL) 14 W fitting E27, Philips 36 W/4 Pin fitting 2G11, Philips PL-C 13 W 2 pin fitting G24, and Philips fluorescent tube 36 W for 4 feet and 18 W for 2 feet. Some of light bulbs are shown in Figure 3.

The Philips liner fluorescent tubes consume 36 W for the 4 feet long linear tube. They have approximately 15000 hours life time and produce 2250 lumens. The 18 W Philips fluorescent tube has lifetime of 15000 hours and produces 1350 lumens. The Philips CFL 14 W bulb comes with E27 fitting. This bulb produces approximately 800 lumens and has life time of 8000 hours. This kind of bulb was used as down light where the light is concentrated in downward direction. The Philips 36 W/4 pin with fitting 2G11 is made up of compact fluorescent tube. Around the campus this kind of tube is commonly used to light up the buildings. These kinds of bulbs can produce up to 2000 lumens and have a life time up to 15,000 hours. The Philips PL-C 13 W 2 Pin fitting G24 produces up to 750 lumens and has life span hours up to 10,000 hours. Use of this bulb is for down lights application. 2 pin designed for easy setting up by just plug and pull.

### 2.1. Ideal Lighting Technology

The fluorescent tube and CFL are the main lighting systems in the campus. However, compared to the new technologies, they are not considered as energy efficient. The proper lighting technology that can be implemented is the LEDs. LEDs use 80% less energy than incandescent and 30% to 40% than most fluorescent lamps. LEDs are environmental friendly where they are mercury free, but fluorescent and CFL contain mercury and required special disposal or recycle method, which contribute to hazardous waste. LED sources have longer lifespans than traditional technologies which can save costs on replacement and maintenance. LEDs offer illumination without emitting harmful infrared or ultraviolet radiation.

**Figure 4:**
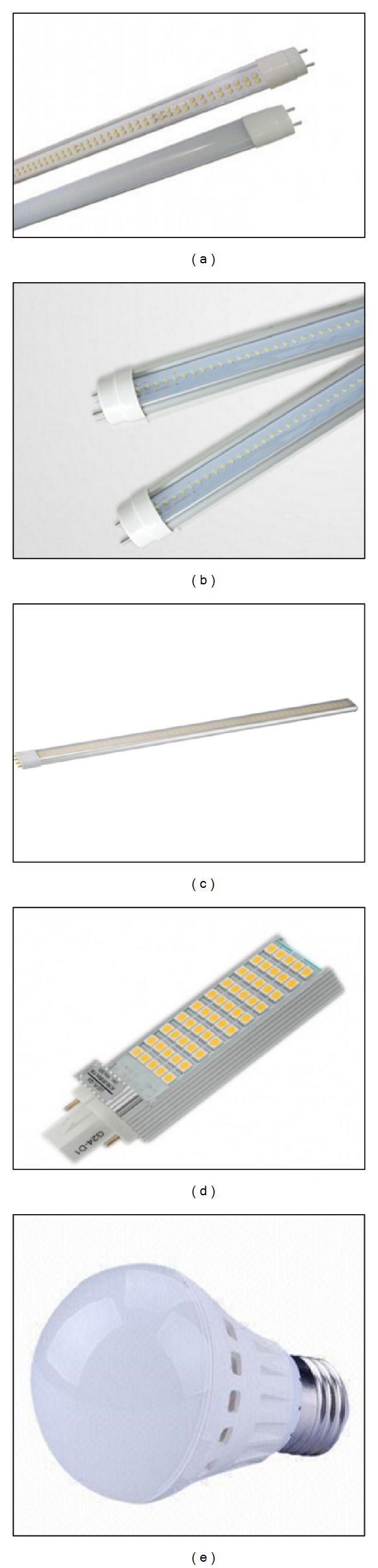
(a) 20 W 4 feet LED tube replacing 36 W fluorescent tube, (b) 10 W 2 feet LED tube replacing 18 W fluorescent tube, (c) 22 W/4 pin LED tube replacing Philips 36 W/4 Pin, (d) 8 W/2 pin LED replacing Philips PL-C 13 W/2 pin, and (e) 9 W LED bulb light replacing Philips 14 W (CFL) bulb.

In current market variable LED tubes are available which offer light output levels similar to 36 W fluorescent tubes. By substituting a 36 W T8 fluorescent tube by a 19 W LED tube can save up to 48%. The wattage and cost of LED tubes to replace 36 W T8s varies according to manufacturers. The advantages of replacing LED tubes are that they are designed to fit directly into current fluorescent fitting by just removing and replacing the starter. LEDs cost higher than T8 to T5 converters but LED tubes have a significantly extensive lifespan of almost five times more dependent on the quality. The types of light chosen to replace the current lighting system are shown in Figure 4. The light retrofitting has been recognized as one of the most effective methods of reducing overall energy consumption as suggested in the ISO50001:2011 standard and recommended practice under the ASEAN Energy Management Scheme (AEMAS). The light bulb selection in this study has been followed by the above mentioned standards to ensure supplying required lighting for staff and students in the selected buildings.

The 4 feet LED tube which consists of 192 LED produces 2100 ± 100 lumens with 50,000 hours lifetime. Efficiency can reach up to 90 lm/W. LED tube has an easy installing method by just removing the current tube including the ballast and simple rewiring which is suggested to be done by qualified electrician. The 2 feet LED tube which is made of 108 LED creates 1150 ± 100 lumens with 50,000 hours life span. The installation method is same as the 4 feet LED tube. The 22 W/4 pin LED tube is made up of 48 pieces SMD5630 LED and produces 1870 ± 100 lumens and also has 50,000 hours lifetime. This tube has a built in driver; therefore, the installation only require removing the electronic ballast of the existing lighting system. The 8 W/2 pin LED is made of 18 pieces SMD5630 LED and produces 650 ± 80 lumens. This LED light has 50,000 hours lifespan and built in driver. Last but not least, the 9 W LED bulb gives an equivalent output of a 14 W CFL. The lumens output is 650 ± 70 lumens and has 30,000 hours lifespan.

### 2.2. Potential Energy Saving

The total daily energy consumption (EC) is calculated by multiplying the total number of lamps (N), power consumed by the lamp (W), and total hours of operation (OH), which is assumed 8 hours. The formula is interpreted in the following equation [10]:

(1) $$\begin{array}{c} \text{EC}=\frac{\left(\text{N}\times \text{W}\times \text{OH}\right)}{1000}. \end{array}$$

Total energy saving (ES) would be the difference between energy consumption of current system (EC_Current_) and the retrofit lighting (EC_Retrofiting_) system:

(2) $$\begin{array}{c} \text{ES}={\text{EC}}_{\text{Current}}-{\text{EC}}_{\text{Retrofiting}}. \end{array}$$

Bill saving (BS) is calculated by the product of energy saving (ES) with electricity tariff (ET). In this case study the electricity tariff is presumed to be increased about 2% every year. Consider

(3) $$\begin{array}{c} \text{BS}=\text{ES}\times \text{ET}. \end{array}$$

### 2.3. Payback Period and Life Cycle Cost Analysis

Payback period (PAY) is the time taken to obtain return of the money that has been invested. In this calculation the present value money is not taken into account during the calculation. Consider

(4) $$\begin{array}{c} \text{PAY}=\;-\frac{\Delta \text{PC}}{\Delta \text{OC}}. \end{array}$$

Life cost analysis (LCC) is the total expenses involved during its lifespan. A standard calculation method for LCC is by summation of failure cost, maintenance cost which is known as the investment cost (PC), and the yearly operation cost (OC) as presented in the following:

(5) $$\begin{array}{c} \text{LCC}=\text{PC}+\left(\text{PWF}\right)\times \left(\text{OC}\right). \end{array}$$

### 2.4. Decrement in Emission

Carbon and Hydrogen are considered as the main constituent of most fuels, followed by a small portion of sulphur. Combustion involves an oxidation reaction, in which the necessary oxygen is usually provided by air, a mixture of oxygen, and nitrogen [19–21]. In Malaysia, natural gas is used as the main fuel for power generation [22]. The emission production (EM) is equal to emission factor (EF) multiplied by the amount of fuel consumed (FC). Therefore, the emission (*p*) due to using fuel (*f*) in the year (*n*) can be calculated with the following [23]:

(6) $$\begin{array}{c} {\text{EM}}_{nf}^{p}={\text{EF}}_{f}^{p}\times {\text{FC}}_{nf}. \end{array}$$

The potential emission production of each fuel based on Malaysian condition is summarized in Table 1 [24].

The predicted amount of emission produced in the process of generating electricity in the years 2014 to 2024 was obtained from the predicted scenarios reported based on local condition [16]. The scenarios are tools for ordering perceptions about alternative future environments, and the result might not be an accurate picture of tomorrow but may give a better decision about the future for policy makers. Regardless of how things can actually be, both the analyst and the decision maker will have a scenario that resembles a given future and will help researchers consider both possibilities and consequences of the future [25, 26]. The result is presented in Table 2.

## 3. Results and Discussion

### 3.1. Lighting Audit

The result of the walk-through lighting audit in the above mentioned selected buildings is presented in Table 3. There are five common types of lighting system used in the selected buildings, which are fluorescent tube 36 W and 18 W, Compact Fluorescent Light (CFL) 14 W, CFL 4 pin 36 W, and CFL 2 pin 13 W. The most commonly used bulb is the Philips 36 W/4 Pin with 2G11 fitting that makes the total number of 28431 bulbs. There are 8751 of 36 W fluorescents 4 feet tube and 12674 of 18 W fluorescent 2 feet tube. The quantity of Philips PL-C 13 W/2 Pin and Philips CFL bulb 14 W are 12719 and 109, respectively. The total number of lamp used in the selected buildings is 62684.

The interview with the staff reveals that in the selected buildings the normal working hours of the lighting system are 8 hours a day, 5 days a week. However, there are always some exceptions due to unscheduled working hours or safety issues.

### 3.2. Energy Consumption

Based on the latest electric tariff rate by the TNB, the national electricity provider, the tariff rate for the low voltage commercial building is RM0.393 per kWh. It was assumed that the electric tariff will have around 2% increase each year [27]. In this survey the policy for retrofitting the old lighting system with the new energy saving LEDs starts with 10% for the first year and continues constantly for 10 years until all the lighting systems have been replaced.

The result for total energy consumption of the existing lighting system is presented in Table 4. The total electricity consumption for the lighting application of the selected buildings is around 13,868.46 kWh per day. Meanwhile, the total consumption of electricity per day for the LED light would be around 8,239.8 kWh that shows the potential 40.59% saving.

Consequently around 1,463,450.56 kWh of energy and RM 517,622 of the bill can be saved each year, if all the existing lighting systems have been replaced with the energy saving LEDs.  Table 5 presents the energy consumption of the proposed LED system in 10 years. It is shown that by increasing the percentage of the retrofitted lighting systems the overall energy consumption decreased. In 2024, when full retrofitting has been achieved the energy consumption will remain constant if the duration of usage of light is supposed constant.

### 3.3. Life Cycle Analysis and Payback Period

To analyze life cycle cost of the lighting system, the total cost of installation, maintenance, and operation of its lifespan have been considered. In this project LCC is used to determine the cost of energy effective progress of LED illumination system which will be implemented. The complete LCC analysis calculation for the 20 W LED tube replaced with the Philips 36 W fluorescent tube is presented in Table 6. The same method have been used to analyze the LCC of 10 W LED tube replaced with Philips 18 W fluorescent tube, 22 W/4 pin LED light replaced with the Philips 36 W/4 Pin, 8 W/2 pin LED bulb replaced with the Philips 13 W/2 pin CFL, and 9 W LED bulb replaced with the Philips 14 W CFL bulb.

**Figure 5:**
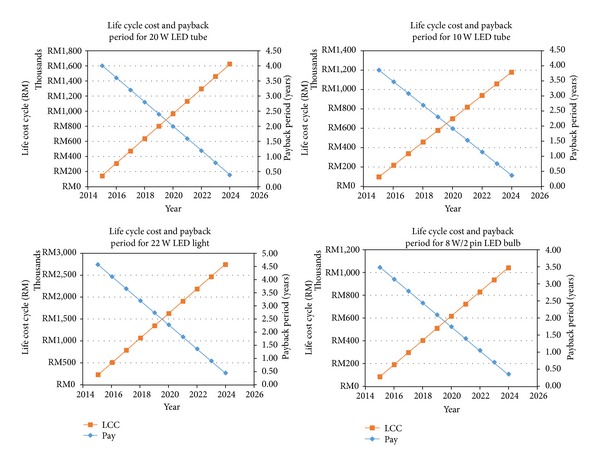
The payback period analysis.

The payback period after complete retrofit for the 20 W LED is 4.01 years, 10 W LED tube is 3.86 years, 22 W/4 pin LED tube is 4.58 years, 8 W/2 pin LED is 3.48 years, and 9 W LED is 4.27 years. Basically after four years the selected buildings will bring profit for the investment. The result of the payback period analysis is shown in Figure 5.

### 3.4. Emission Reduction

**Figure 6:**
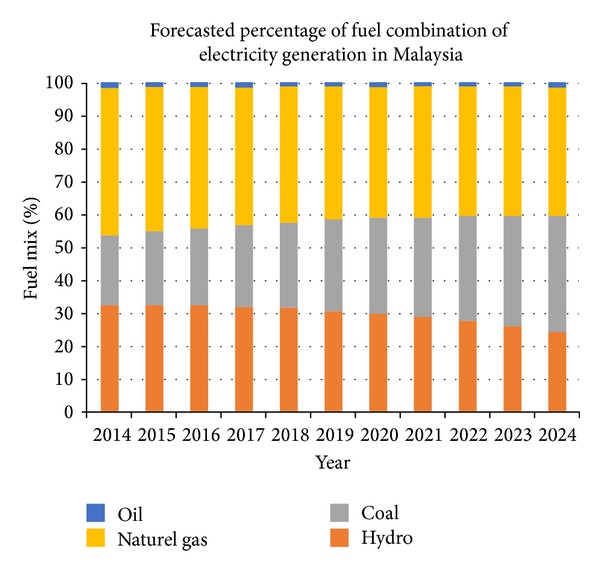
Forecasted percentage of fuel combination of electricity generation in Malaysia.

Analysis on emission reduction by retrofitting energy saving LEDs is conducted by predicting the future data of fuel mixing to generate electricity in Malaysia. However, the used data might have variance with the real condition due to the uncountable reasons that are not predictable and is out of scope of this study. The predicted fuel combination to generate electricity in Malaysia that is used in this project is presented in Figure 6. As the graph shows, Malaysia is also beginning to invest toward renewable energies.

**Figure 7:**
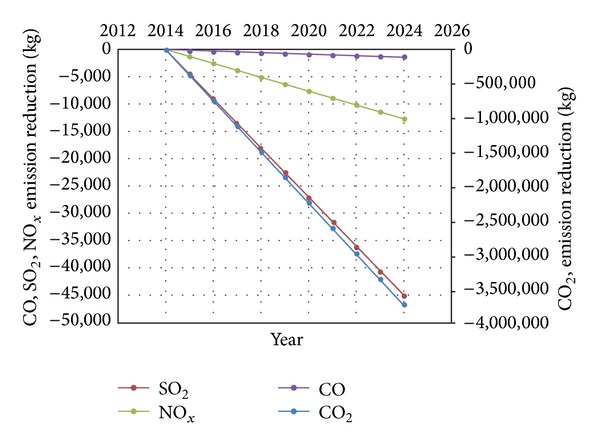
Pattern of emission reduction of CO_2_, SO_2_, NO_*x*_, and CO by electricity generation from 0% to 100% retrofitting.

As a result of retrofitting, the overall amount of electricity consumption decreases and consequently would help the environment by decreasing the harmful effect of the greenhouse gases. The proposed model suggests overall emission production reduction of around 3,746,433 kg of CO_2_, 23,473 kg of SO_2_, 12,585 kg of NO_*x*_, and 1,317 kg of CO after 100% retrofit. Graph of total reduction in gases emission of CO_2_, SO_2_,  NO_*x*_, and CO by electricity generation from 0% to 100% retrofitting is presented in Figure 7, which shows the increment of reduction in emission every year.

## 4. Conclusion

Day by day the usage of electricity increases globally due to the ever increasing demand in the developed and developing countries. One of the fast options to save the energy is to use energy efficient electrical appliances among which lighting system has a great potential on saving energy. The present project has focused on the selected buildings in the Universiti Tenaga Nasional campus with the objective of designing a proper retrofit scenario and calculates the potential electricity saving, the payback period, and the potential environmental benefits. In this project, the energy saving and emission production reduction have been analyzed based on the comparison between the existing lighting system and the proposed LED retrofitting. In this survey the policy for retrofitting the old lighting system with the new energy saving LEDs starts with 10% for the first year and continues constantly for 10 years until all the lighting systems have been replaced. The result of the life cycle analysis reveals that after four years, the selected buildings will bring profit for the investment. Further comparison has been done between T5 electronic ballast and LED tube. Both T5 and LED are much more energy efficient compared to the existing CFL lighting system. By retrofitting 100% of the existing lighting system with the LED lights, around 44% saving of energy can be saved with payback period of 4.01 year. However, by using T5 tube with electronic ballast the saving limits to 22% with payback period of 3.8 years. Although, the initial cost of LED lighting system is higher compared to T5 electronic ballast, it would bring more saving in long term.

## Figures and Tables

**Table 1:** Emission factors (kg/kWh) used for estimating emission in power plants.

Fuel type	CO_2_	NO_*x*_	SO_2_	CO
	Kg/kWh			
Coal	1.18	0.0052	0.0139	0.0002
Natural gas	0.53	0.0009	0.0005	0.0005
Fuel oil	0.85	0.0025	0.0164	0.0002
Diesel	0.85	0.0025	0.0164	0.0002

**Table 2:** The amount of CO_2_, SO_2_, NO_*x*_, and CO emission production per kWh of electricity generation.

Fuels	Emission (kg/kWh)			
	CO_2_	SO_2_	NO_*x*_	CO
Coal	1.1800	0.0139	0.0052	0.0002
Petroleum	0.8500	0.0164	0.0025	0.0002
Gas	0.5300	0.0005	0.0009	0.0005

**Table 3:** Number of light collected for each building.

Buildings	Types of light used				
	Fluorescent tube 36 W 4 feet	CFL 14 W	13 W 2 pin	2 pin CFL 36 W	Fluorescent tube 18 W 2 feet
BA Admin	396	0	2372	5136	93
BB	5	14	244	816	8
BC	2	10	404	888	4
BD	20	11	124	204	4
BE	46	18	296	924	4
BF	44	10	200	102	4
BG	2	12	336	1122	4
BH	10	10	248	288	4
BJ	18	12	270	924	4
BL	56	0	51	2154	36
BM	105	0	786	1929	38
BN	61	0	335	3609	27
BV	8	0	0	252	135
Food court	108	12	0	0	0
COIT BW	286	0	3212	4989	97
Amanah	1933	0	605	0	1824
Murni	1320	0	0	0	3698
Ilmu	1893	0	612	0	1812
Cendekiawan	1056	0	0	0	4752
Library	1382	0	2624	5094	126

Total	8751	109	12719	28431	12674

**Table 4:** Energy consumption between present and alternative light per day.

Present Light	Fluorescent tube 36 W 4 feet	CFL 14 W	13 W/2 pin	CFL 36 W/4 pin	Fluorescent tube 18 W 2 feet	Total (kWh)
kWh	2,520.29	12.21	1,322.78	8,188.13	1,825.06	13,868.46
Alternative light	20 W-4 ft LED tube	9 W LED bulb	8 W/2 pin LED	22 W/4 pin LED tube	10 W-2 ft LED tube	Total (kWh)
kWh	1,400.16	7.85	814.02	5,003.86	1,013.92	8,239.80

**Table 5:** Energy consumption between existing and alternative fixtures.

Year	LED energy consumption (kWh)
2014	3,605,798.56
2015	3,459,453.50
2016	3,313,108.45
2017	3,166,763.39
2018	3,020,418.34
2019	2,874,073.28
2020	2,727,728.22
2021	2,581,383.17
2022	2,435,038.11
2023	2,288,693.06
2024	2,142,348.00

**Table 6:** LCC analysis for the 20 W LED tube replacing Philips 36 W fluorescent tube.

Variables	Value	Unit
LED-wattage	20	Watts
LED-single unit cost	RM60.00	Ringgit
Existing light-wattage	36	Watts
Existing light-single unit cost	RM10.00	Ringgit
Number of lamps	8751	Lamp(s)
Running time per day	8	Hours
Operational days (per year)	260	Days
Cost of energy/kWh	RM0.39	Ringgit

Calculations	Existing system	LED

Electrical costs		
Electrical load of lamp(s)	315036	175020
Running time per year	2080	2080
Energy consumed per year (kWh)	655274.88	364041.6
Electrical demand saving (kWh)	0	291233.28
Total cost of energy (per year)	RM257,523.03	RM143,068.35
Saving the electrical fees (per year)	RM0.00	RM114,454.68
Capital requirements		
Purchase requirements Cost	RM87,510.00	RM525,060.00
Installation cost per unit	RM2.00	RM1.00
Installation costs	RM17,502.00	RM8,751.00
Total capital investment requirements	RM105,012.00	RM533,811.00
Net investment requirement	RM0.00	RM428,799.00
Maintenance requirements		
Life time of lamp (operating hours)	15000	50000
Replacements required (per year)	1213.47	364.04
Replacement costs (per year)	RM12,134.72	RM21,842.50
Installation cost per new unit	RM2.00	RM1.00
Maintenance costs (per year)	RM2,426.94	RM364.04
Total maintenance costs (per year)	RM14,561.66	RM22,206.54
Saving maintenance (per year)	RM0.00	−RM7,644.87
ROI results		
Total operating cost (per year)	RM272,084.69	RM165,274.89
Total savings first year Saving the electrical fees + savings maintenance	RM0.00	RM106,809.81
Payback period in years Time to payback capital requirement cost	n/a	4.01
LED return on investment (ROI) over 10 years	RM0.00	RM534,287.05

## References

[1] Uddin S, Shareef H, Mohamed A (2013). Power quality performance of energy-efficient low-wattage LED lamps. *Measurement*.

[2] Soori PK, Vishwas M (2013). Lighting control strategy for energy efficient office lighting system design. *Energy and Buildings*.

[3] Mahlia TMI, Said MFM, Masjuki HH, Tamjis MR (2005). Cost-benefit analysis and emission reduction of lighting retrofits in residential sector. *Energy and Buildings*.

[4] Trifunovic J, Mikulovic J, Djurisic Z, Djuric M, Kostic M (2009). Reductions in electricity consumption and power demand in case of the mass use of compact fluorescent lamps. *Energy*.

[5] Ma Z, Cooper P, Daly D, Ledo L (2012). Existing building retrofits: methodology and state-of-the-art. *Energy and Buildings*.

[6] Lee AHW (2000). Verification of electrical energy savings for lighting retrofits using short- and long-term monitoring. *Energy Conversion and Management*.

[7] Caicedo D, Pandharipande A, Leus G (2011). Occupancy-based illumination control of LED lighting systems. *Lighting Research and Technology*.

[8] Choi S, Kim T (2012). Symmetric current-balancing circuit for LED backlight with dimming. *IEEE Transactions on Industrial Electronics*.

[9] Li Y-C, Chen C-L (2012). A novel single-stage high-power-factor AC-to-DC LED driving circuit with leakage inductance energy recycling. *IEEE Transactions on Industrial Electronics*.

[10] Mahlia TMI, Razak HA, Nursahida MA (2011). Life cycle cost analysis and payback period of lighting retrofit at the University of Malaya. *Renewable and Sustainable Energy Reviews*.

[11] Di Stefano J (2000). Energy efficiency and the environment: the potential for energy efficient lighting to save energy and reduce carbon dioxide emissions at Melbourne University, Australia. *Energy*.

[12] Uddin S, Shareef H, Mohamed A, Hannan MA, Mohamed K LEDs as energy efficient lighting systems: a detail review.

[13] Chen N, Chung HS-H (2011). A driving technology for retrofit LED lamp for fluorescent lighting fixtures with electronic ballasts. *IEEE Transactions on Power Electronics*.

[14] Ryckaert WR, Smet KAG, Roelandts IAA, van Gils M, Hanselaer P (2012). Linear LED tubes versus fluorescent lamps: an evaluation. *Energy and Buildings*.

[15] Vahl FP, Campos LMS, Casarotto Filho N (2013). Sustainability constraints in techno-economic analysis of general lighting retrofits. *Energ Buildings*.

[16] Mahlia TMI (2002). Emissions from electricity generation in Malaysia. *Renewable Energy*.

[17] Pei-Dong Z, Guomei J, Gang W (2007). Contribution to emission reduction of CO_2_ and SO_2_ by household biogas construction in rural China. *Renewable and Sustainable Energy Reviews*.

[18] Mahalingam E (2010). *New Green Rating Tools to Boost Value of Old Buildings*.

[19] Heisler H (1995). *Advanced Engine Technology*.

[20] Johnson AJ, Auth GH (1951). *Fuels and Combustion Handbook*.

[21] Wallace FJ, Linning WA (1970). *Basic Engineering Thermodynamics: S.I. Units*.

[22] Mazandarani A, Mahlia TMI, Chong WT, Moghavvemi M (2010). A review on the pattern of electricity generation and emission in Iran from 1967 to 2008. *Renewable and Sustainable Energy Reviews*.

[23] Shekarchian M, Moghavvemi M, Rismanchi B, Mahlia TMI, Olofsson T (2012). The cost benefit analysis and potential emission reduction evaluation of applying wall insulation for buildings in Malaysia. *Renewable and Sustainable Energy Reviews*.

[24] Shekarchian M, Moghavvemi M, Mahlia TMI, Mazandarani A (2011). A review on the pattern of electricity generation and emission in Malaysia from 1976 to 2008. *Renewable & Sustainable Energy Reviews*.

[25] (1996). *P S. The Art of the Long View: Planning in an Uncertain World*.

[26] Mazandarani A, Mahlia TMI, Chong WT, Moghavvemi M (2011). Fuel consumption and emission prediction by Iranian power plants until 2025. *Renewable and Sustainable Energy Reviews*.

[27] Tenaga Nasional Sdn Bhd

